# Combining market and nonmarket food sources provides rural households with more options to achieve better diets in Southern Benin

**DOI:** 10.1007/s12571-022-01320-w

**Published:** 2022-11-25

**Authors:** Mauricio R. Bellon, Gervais Ntandou-Bouzitou, Janet E. Lauderdale, Francesco Caracciolo

**Affiliations:** 1 Swette Center for Sustainable Food Systems, Arizona State University, PO Box 878204, 85287-8204 Tempe, AZ USA; 2 Food and Agriculture Organization (FAO) of the United Nations, Niamey, Niger; 3 Tempe, USA; 4 Department of Agricultural Sciences, University of Naples Federico II, Portici (Na), Italy

**Keywords:** Production diversity, Market participation, Dietary diversity, Benin, West Africa

## Abstract

This study examines the relative contributions to dietary diversity of the diversity of plant and domesticated animal species which rural households produce or collect, i.e., nonmarket food sources, versus the diversity of foods purchased in markets. Although opinions differ in the literature as to their relative importance, clarifying how different sources of food contribute to the dietary diversity of rural households in the developing world is important to inform policies and interventions to improve their food security and dietary quality. This case study was carried out among a random sample of 654 rural households in southern Benin during two seasons: when food is plentiful after harvest; and when food is scarcer between harvests. We collected data on crops, wild plants, and domesticated animals utilized by households, the number of markets they visited, and the diet of a mother in the household, with a structured 24-hour food frequency questionnaire. We hypothesize that the number of markets visited is an indicator of the diversity of foods available in the markets they have access to, and thus shows the contribution of markets as food sources. Results support this hypothesis and show that households that produced more plant and domesticated animal species and those that visited more markets had more diversified diets. Obtaining diverse foods from multiple sources provides households with more options to achieve better diets. These results suggest a need for a more holistic approach that recognizes the complementarities between market and nonmarket sources of foods. This approach should build on the diversity of species rural households already utilize, and on the ways they interact with markets.

**Supplementary Information:**

The online version contains supplementary material available at 10.1007/s12571-022-01320-w.

## Introduction

Improving the nutrition and food security of rural populations in developing countries is an important part of addressing Sustainable Goal 2 (Zero Hunger). Dietary diversity (DD) is universally recognized as a key component of healthy diets and is strongly associated with nutrient adequacy (Arimond et al., 2010; Ruel, 2003), as well as being an indicator of food security (Jones et al., 2013). There is a recent and growing literature examining the contribution of local crops grown and wild species gathered by rural households (HHs) in various parts of the developing world, generally referred to as production diversity (PD), to their diets and food security (Jones, 2017a; Sibhatu & Qaim, 2018). An important policy debate emerging from this literature is the relative merits of supporting PD for self-consumption versus encouraging improved market participation and crop specialization by HHs as ways to improve their dietary diversity (DD) and food security. Empirical evidence, mostly based on regression analyses, shows a positive association between the PD managed by rural HHs and the DD they consume as a unit or by some of their members (usually mothers and/or children), though results are context specific (Bellon et al., 2016; Dillon et al., 2015; Ecker, 2018; Fraval et al., 2020; Jones et al., 2014; Jones, 2017b; Hirvonen & Hoddinott, 2017; Koppmair et al., 2016; Muthini et al., 2020; Powell et al., 2015; Sibhatu et al., 2015; Zanello et al., 2019). These findings have led some researchers to argue for the need to encourage policies and initiatives promoting PD for self-consumption to improve HH dietary quality and food security (Jones et al., 2014; Powell et al., 2015; Remans et al., 2015). However, there is a clear recognition that rural HHs purchase a large proportion of the foods they consume in markets (Bellon et al., 2016; Hirvonen & Hoddinott, 2017; Muthini et al., 2020). This has led other researchers to argue that promoting better participation of rural HHs with markets may be a more effective way to improve dietary quality and food security (Dillon et al., 2015; Sibhatu et al., 2015; Hirvonen & Hoddinott, 2017). It is claimed that enhanced market participation of HHs can lead to higher incomes allowing them to purchase and consume a higher quantity and diversity of foods, leading to better diets. Therefore, interventions or policies promoting market participation of smallholder farmers may be more effective than those promoting increases in PD for improving HH nutrition (Koppmair et al., 2016; Hirvonen & Hoddinott, 2017; Muthini et al., 2020; Sibhatu et al., 2015; Sibhatu & Qaim, 2018). In addition, it is asserted that promoting diversification for the sake of encouraging PD for self-consumption can entail important costs by preventing gains from specialization that could result in less income leading to negative nutritional effects (Koppmair et al., 2016; Muthini et al., 2020; Sibhatu et al., 2015; Sibhatu & Qaim, 2018).

The argument for prioritizing market access over PD for improving diets is often based on a simplistic unidirectional conceptualization of the relationships among PD, DD, and market participation. This argument has a number of flaws. First, the PD that HHs maintain cannot necessarily be considered an independent decision from the DD they eat, since consumption considerations may influence production choices to the extent that an important objective of agricultural production among smallholder farmers is self-consumption (Jones, 2017b; Ecker, 2018; Muller, 2009). This means that one cannot assume a unidirectional causality from PD to DD. Thus, regression analyses that estimate this relationship without correcting for the potential influence of DD on PD, referred as endogeneity, may be incorrect. Second, the variety of foods purchased by farming HHs and their PD are generally interconnected decisions as well, involving dietary substitution, i.e. if a certain food item is produced by the HH, it does not need to be purchased (Muthini et al., 2020). Third, certain foods may not be available in markets and the only way to obtain them may be either by self-producing or collecting them (Muller, 2009; Powell et al., 2015). Fourth, in much of the literature, markets have been treated as a “black box” (Ickowitz et al., 2019) and market access and participation have been mainly measured in overly simplified ways (Verger et al., 2021). For the most part, market access has been measured by some simple measure of travel distance or time to a market (Jones, 2017b; Koppmair et al., 2016; Hirvonen & Hoddinott, 2017; Sibhatu et al., 2015), or the cost of transporting a product to a market (Zanello et al., 2019). Measures of market participation include the presence or share of crops sold (Koppmair et al., 2016; Sibhatu et al., 2015); the area planted with non-food crops (Koppmair et al., 2016); and the presence of off-farm income (Sibhatu et al., 2015). These measures ignore the specific availability, affordability, convenience and desirability of food, i.e., the food environment, faced by HHs (Herforth & Ahmed, 2015). Fifth, seasonality, a common and crucial factor for rural HH farming under rainfed conditions, has not always been considered in studies on the relationship between PD and DD (M’Kaibi et al., 2015; Zanello et al., 2019). So, clearly the argument for prioritizing improved market participation and crop specialization to improve the diets of rural HHs is based for the most part on studies with simplistic views of the interactions among PD, DD, and markets, and ways to measure them.

This paper aims to contribute to this debate by presenting a case study among rural HHs in southern Benin, West Africa, to assess the contributions of their participation in markets and of the PD they maintain to the sources of foods that underpin the DD they eat during two seasons: when food is plentiful after the main harvest; and when food is scarcer as stores are depleted between harvests. We distinguish two broad types of food sources: market and nonmarket. The former comprises the diversity of foods HHs purchased, while the latter includes the crops, wild and semi wild plant, and domesticated animal species they produce or gather for self-consumption, as well as food gifts, referred to here as self-provisioning. We included gifts since they reflect reciprocity and a moral dimension of food sourcing that can be important in African rural settings (Adams, 1993). Food aid is insignificant in our study area and was ignored.

We test for the effects of households’ production diversity and of market participation on the dietary diversity they consume derived from foods purchased and foods produced for self-consumption (market and nonmarket sources respectively). Given that the contribution of market participation by a household to its dietary diversity rests on the diversity of available foods that can be purchased in markets and their affordability (Gelli et al., 2020; Herforth & Ahmed, 2015; Zanello et al., 2019), we hypothesize that the number of markets visited by households is an indicator of the diversity of foods available in the markets they have access to. The reason is that going to a market is costly in terms of transaction and opportunity costs (Barrett, 2008). Since each additional market visited increases these costs, a household’s willingness to incur them should be related to the likelihood of acquiring additional foods demanded given its income and location, which in turn should be related to the distribution of diverse foods across available markets. In other words, why would someone incur the cost of going to a second market if she can get everything she wants and can afford in one market? Only if she cannot find what she wants in the first market. While one would expect a positive effect of production diversity on dietary diversity derived from production for self-consumption, it is not clear how the latter may be influenced by market participation. Nor how production diversity may influence the dietary diversity derived from purchased foods. Determining the significance and direction of these multiple impacts is important to understand the trade-offs and synergies that different food sources have on households’ dietary diversity, and that is at the core of the debate reviewed above.

This study is a follow-up to earlier work where we showed that PD and the number of foods purchased by rural HHs in Benin are positively associated with DD (Bellon et al., 2016). Here, we provide a more in-depth picture of HHs’ market participation for sourcing food and its contribution to different food groups. This expands on our previous paper where the relative importance of the variety and nutritional contribution of purchased food items was not adequately considered (Verger et al., 2021). As in our previous study, we use HH-level data for PD, market participation and other socioeconomic factors, but focus on women of reproductive age (mothers) for food consumption and dietary diversity as representative of the HH. Within the HH, women are responsible for preparing meals and rearing children and as such their decisions affect the whole HH (Richards et al., 2013; Savy et al., 2005). Furthermore, for the most part male and female HH members eat similar food dishes from the same pot and thus have similar food sources. If there are gender differences in food allocation, focusing on HH women provides a conservative view of HH food consumption and DD if they consume lower diversity and amounts of foods than other HH members. However, studying intra-household food allocation is beyond the scope of this study.

## Methods and data

The present study took place in three districts in southern Benin, West Africa. The districts were selected to represent different levels of urbanization characterized by varying ratios of rural versus urban population densities within a similar agroecological environment. The objective was to select a sample of rural HHs living in districts with different ratios of urban vs. rural population densities since we assume that variation in these ratios indicates different opportunities for market participation for its inhabitants. The districts are in the Departments of Atlantique and Zou and included: (1) Bohicon, a district with a higher urban vs. rural population density, referred here as “urban”; (2) Allada a district with higher rural vs. urban population density, referred as “semi-urban”; and (3) Toffo, a district with an entirely rural population, referred as “rural.” Data on urban and rural population densities were obtained from HarvestChoice, IFPRI, University of Minnesota (2017). Within each district, villages were randomly selected within three bands of travel time to the main town in the district, showing different levels of accessibility (high: ≤ 30min, medium: 31–59min, low: ≥ 60min). A total of 33 villages were selected, and within each of them, 15 HHs with a mother (15–49 years old) and a child (6–59 months old) were randomly chosen. Notwithstanding the different types of districts, only rural HHs were selected within them. Two rounds of interviews took place to account for seasonality. In the study area there are two rainy seasons when crops are grown: the short rainy season, from mid-September to early December, and the long rainy season, from March to the end of July (INSAE, 2016a, b). The first round of surveys, which included 472 HHs, took place from November to December 2010, at the end of the short rainy season, when crops of that season have just been harvested and there is plenty of food available (abundance period). The second round that included 482 HHs, took place April to May 2012, during the long rainy season, when crops have been planted but not yet harvested, and food from most of these crops is still not available (scarcity period), though some foods derived from short-duration crops or from collecting wild and semi-wild species may be accessible. In total our sample consisted of 652 HHs, with 302 HHs in common and a pooled sample of 954 observations. A detailed explanation of the sampling strategy and the calculations for sample sizes is presented in Bellon et al. (2016)

The surveys elicited information from the mother and father in a HH on general socioeconomic characteristics of HHs, such as ethnicity, language, age and formal education of the mother and father, their respective sources of farm and non-farm incomes and their importance, size of landholdings, and ownership of household and productive assets. We obtained a list of all useful plant species grown and collected, as well as domesticated animal species managed by the mother and the father in a HH during the most recent agricultural season to each of the two rounds of surveys that were carried out. Gathered plants were included since they have been shown to contribute to rural diets and can be particularly important for HHs without land (Powell et al., 2015). Based on this list, counts of plant species grown and collected, as well as domesticated animal species, were calculated and used as indicators of PD at the HH level. We did not collect information on hunted fauna.

To characterize market participation, the mother and father were asked about all the markets that they visited in the 15 days prior to the interview; including the names of the locations visited, travel time to each of them, and frequency of their visits. Markets were defined and identified by the respondents as places they visit to sell or purchase foods and other goods. To estimate the total amount of time HHs invested for visiting markets during the 15-day period, travel time to each market was multiplied by two (round trip) and by the frequency of visits, and then added for all markets visited. Although this number does not include the time spent in the market and the monetary costs of the visit, it provides a good approximation of the costs of market participation.

Qualitative food consumption data of the mother in a HH were collected through a structured food frequency questionnaire over a 24-hour recall period. Data on all food items consumed were recorded in a completely disaggregated way, including different preparations of the same species, and the source of the foods consumed: self-production, purchase, gathering, gifts, borrowing and food aid. All foods consumed were classified into 16 food groups from the FAO Guidelines for Measuring HH and Individual Diversity (Kennedy et al., 2010) by the source from which they were obtained. These food groups are a good proxy for a HH’s economic access to a variety of foods (Kennedy et al., 2010). While usually these 16 food groups are aggregated into 12 food groups to calculate the HH dietary diversity score (HDDS), here we kept the original number without aggregation to provide a more comprehensive perspective of the contributions of different types of foods to the dietary diversity. From these data, two dietary diversity scores were compiled for use in the empirical analysis. One by counting the number of food groups provided by purchased foods, referred as dietary diversity from purchases, i.e., from the market (DD_P_). The other from the count of food groups provided by the combination of three nonmarket sources of foods: production for self-consumption, gathering of wild and semi-wild species and food gifts, referred here as dietary diversity from self-provision (DD_S_).

To analyze the complexity of the relations among HHs’ production diversity (PD) and market participation (MP) on the two mother’s dietary diversity components, we used a similar approach as in our previous paper (Bellon et al., 2016) adapted to the specific purposes of this study. We estimated a system of four simultaneous equations via a Generalized Method of Moments (Hayashi, 2000) formulated as follows for the *i*-th HH and associated mother:

1 $$P{D_i}\, = \,exp\left( {{\bf{x_i}}{^\prime }{\boldsymbol{\beta}} \, + \,{\bf{z_i}}{^\prime }\alpha } \right)\, +{\text{e}_{i}}$$

2 $$M{P_i}\, = \,exp\left( {{\bf{x}}_{\text{i}}{^\prime }{\boldsymbol{\lambda}} \, + \,{\bf{z}}_{\text{i}}{^\prime }{\boldsymbol{\pi}}} \right)\, + \,u_i$$

3 $$DD{{\text{s}}_i}\, = \,exp\left( {{\bf{x}}_{\text{i}}^\prime {\boldsymbol{\theta}} \, + \,\xi P{D_{\text{i}}}\, + \,\nu M{P_i}} \right)\, + \,\varepsilon_i$$

4 $$D{D_P}_{\text{i}}\, = \,exp\left( {{\bf{x}}_{\text{i}}^\prime {\boldsymbol{\tau}} \, + \,\delta P{D}_{i}\, + \,\phi M{P_i}} \right)\, + \,\nu_i$$

The first two equations model the factors (PD and MP) that we hypothesize influence the sources of dietary diversity and correct for reverse causality (endogeneity) that may exist between these factors and the outcomes of interest. The other two equations allow to test the hypotheses about the effects of PD and MP on the outcomes of interest (DD_s_ and DD_p_), while correcting for endogeneity. In all four equations, we included other variables that could influence the factors of interest to correct for their potential effects on the relationships being tested, i.e., covariates.

We used a Poisson-based specification for the four equations since in all cases the dependent variables represent count measures of plant species, markets, and food categories (Windmeijer & Santos Silva, 1997) (further technical details on the estimation are provided in Supplemental Material, Text 1). To address the issue of endogeneity, (i.e., simultaneity, reverse causality between the dependent and independent variables in Eqs.1–4), we follow the identification strategy described in detail in Bellon et al. (2016), with the size of landholdings, and wealth, operationalized as a socio-economic status (SES) index, as instrumental variables. An instrumental variable is correlated with an independent variable but not with the dependent variable, allowing for the estimation of the causal effect of the former on the latter. The covariates and exogenous variables were the same as those used in Bellon et al. (2016) (Table1).

**Table 1:** Definition of variables used in the econometric model

Variable names	Variable definitions
**Dependent variables**	
Production diversity (PD)	Number of cultivated, wild and semi-wild plant species grown and collected, and domesticated animals maintained by a HH (mother and father)
Market participation (MP)	Number of markets visited by a HH (mother and/or father) in the previous 15 days
DD from self-provision (DD_S_)	Number of food groups (out of 16) consumed by mother in the HH sourced from self-provisioning (grown, gathered of received as gifts) from the last 24-hour recall
DD from purchases (DD_P_)	Number of food groups (out of 16) consumed by mother in the HH sourced from purchases from last 24-hour recall (dietary diversity from the market)
**Instrumental variables**	
Landholdings	HH landholdings (ha)
Square of landholdings	Squared of HH landholdings (ha^2^)
Socioeconomic Index	Index of socioeconomic status, predicted 1st factor from a Factor Analysis performed on the number of bicycles, motorcycles, motor vehicles, radios, TVs, and cell phones owned by a HH, calculated for all HHs in both seasons together
**Covariates**	
Travel time	Travel time to main market town in the relevant district (minutes)
Square of travel time	Square of the travel time (minutes^2^)
No. non-agricultural income sources -Father	Number of sources of income besides own agriculture of father, indicator of participation in non-agricultural economy
Agriculture rated very important income source-Father	Dummy of whether agricultural sources of income were rated as very important by father (1 = yes, 0 = no)
Agriculture rated important income source-Father	Dummy of whether agricultural sources of income were rated as important by father (1 = yes, 0 = no).
No. non-agricultural income sources -Mother	Number of sources of income besides own agriculture of mother, indicator of participation in non-agricultural economy
Agriculture rated very important income source-Mother	Dummy of whether agricultural sources of income were rated as very important by mother (1 = yes, 0 = no)
Agriculture rated important income source-Mother	Dummy of whether agricultural sources of income were rated as important by mother (1 = yes, 0 = no).
Mother age	Age of mother (years)
Mother education	Number of years of formal schooling completed by mother
Mother ethnicity	Ethnicity of mother (1 = Aizo, 0 = other)
Family size	Family size, number of family members
Urban district	Dummy indicating if the HH lives in in the “urban” district (1 = yes, 0 = no)
Semi-urban district	Dummy indicating if the HH lives in a in the “semi-urban” district (1 = yes, 0 = no)
Season	1 = abundance period, November to December 2010; 0 = scarcity period April to May 2012

We carried out diagnostic tests for endogeneity and the appropriateness of the variables used as instruments.

The study was approved by the National Ethics Committee of Benin. The subjects, as well as heads of selected HHs and local authorities, were informed of the purpose and procedures of the study. All participants signed an informed consent form. Socioeconomic data were collected through face-to-face interviews with mothers (in collaboration with their husbands) using a semi-structured questionnaire.

## Results

### Market participation

HHs visited 42 and 36 markets during the periods of abundance and of scarcity respectively, with an overall total of 55 different markets. These markets can be grouped into four types: primary; rural consumer; rural assembly; and regional (Honfoga et al., 2018). Primary markets are local markets that take place once or twice a week. Farmers sell directly to village retailers and small-scale assemblers, mostly in small quantities. Rural consumer markets are district-level retail markets that take place every day and have permanent retailers, but also itinerant traders, and once or twice a week farmers may sell wholesale directly there as well. Rural assembly markets are district-level markets that take place two or three times a week where products from many villages/primary markets are assembled for rural semi-wholesalers, but retail consumers can purchase products as well. Regional markets collect products from assembly markets for urban wholesalers; they take place every day and have permanent traders, as well as itinerant traders, and once or twice a week farmers may sell wholesale directly there as well. In some towns rural consumer and regional markets coincide.

These types provide different trading opportunities and levels of product diversity (additional information presented in Supplemental Material, Text 2). In both seasons, rural consumer markets were the most visited, followed by regional, primary, and rural assembly markets, with some variation among districts (Supplemental Material Table S.1). The number of markets visited varied among districts during both periods, with the lowest number by HHs in the urban district in both periods and the highest number by HHs in the rural district during the period of scarcity. On average, HHs visited more than one market, with differences among districts and periods (Table2).

Total traveling time was greater during the scarcity period, indicating a higher reliance of HHs on markets to access foods. There were differences in both periods across districts, with shorter times in the urban district compared to the other two. For HHs that went to markets during both periods, there is a positive relationship between the natural logarithm of travel time they invested, and the number of markets visited, with positive correlations of 0.47 (p < 0.0001) and 0.45 (p < 0.0001) for the abundance and scarcity periods respectively (Supplemental Material Fig.S1). These results confirm that visiting more markets is costly and these costs are not linear, but increase exponentially, since the relationship is logarithmic.

**Table 2:** Markets visited by mothers by period and district

	Abundance				Scarcity			
	Urban	Semi-Urban	Rural	All	Urban	Semi-Urban	Rural	All
Number of villages	9	11	13	33	9	11	13	33
No. of HHs	115	174	183	472	115	176	191	482
No. of markets	14	17	16	42	12	13	26	36
Mean no. of markets visited by a HH	1.24^a^ (0.56)	1.56^b^ (0.68)	0.98^c^ (0.83)	1.26 (0.76)	1.40^a^ (0.75)	1.56^a,b^ (0.66)	1.26^a,c^ (0.58)	1.40 (0.66)
Total amount of traveling time^2^	157.2^a^ (138.1)	264.0^b^ (307.0)	294.7^b^ (435.7)	244.5 (327.1)	236.7^a^ (221.3)	359.5^b^ (346.9)	308.7^a,b^ (418.7)	310 (356.2)

^1^ Standard deviation in parenthesis

^2^Number of minutes to reach the market multiplied by 2 (roundtrip) multiplied by the number of visits per market and the number of markets visited, only for mothers that visited at least one market during the 15-day period previous to the day the survey took place

^a, b,c^ Mean values across a row for a period (abundance, scarcity) with different superscripts letters were statistically significantly different (P = 0.05) using a Scheffé multiple-comparison test after a One way ANOVA with district as a factor

### Production diversity

During the agricultural season associated with the abundance period (short rainy season), 75.6% of HHs cultivated, collected and/or maintained a total of 42 species of plants and 7 domesticated animal species, with an average per HH of 2.9 (± 3.1) crop species, 0.4 (± 1.0) wild or semi-wild species and 1 animal species (± 1) for a combined 4.3 (± 4) species. During the agricultural season associated the scarcity period (long rainy season), this increased to 95.2% of HHs with a total of 64 species of plants and 10 domesticated animal species, with an average per HH of 4.4 (± 3.4) crop species, 1.8 (± 1.8) wild or semi-wild species and 1.3 (± 1.1) animal species for a combined 7.5 (± 4.3) species.

### Sources of mothers’ dietary diversity

Mothers consumed on average 6.6 (± 1.8) and 7.7 (± 1.7) food groups during the 24-hour recall period for the abundance and scarcity periods respectively. Most of the food groups were purchased (4.8 ± 1.7 and 6.3 ± 1.9), followed by self-produced (2.1 ± 1.3 and 1.5 ± 1.5) with gathering and gifts contributing very little on average. However, a significant percentage of mothers obtained certain food groups by gathering or through gifts. For example, for the abundance period, 61.9%, 60%, 85.1% and 69.6% of mothers that consumed dark-green leafy vegetables, other fruits, vitamin A-rich fruits, and meat/poultry respectively, obtained them from gathering or gifts; while for the scarcity period, it was 45.6% and 45.5% of mothers consuming dark-green leafy vegetables and vitamin A-rich fruit respectively. So, while gathering and gifts were not important sources for the overall dietary diversity, they made a very important contribution to some specific nutritionally relevant food groups during both periods. Purchases were always an important source for most food groups, but particularly dominant for fish/seafood, legumes/nuts/pulses, cereals, fats and oils, and spices and condiments and drinks in both periods (Supplemental Material Tables S2 and S3).

**Fig. 1:**
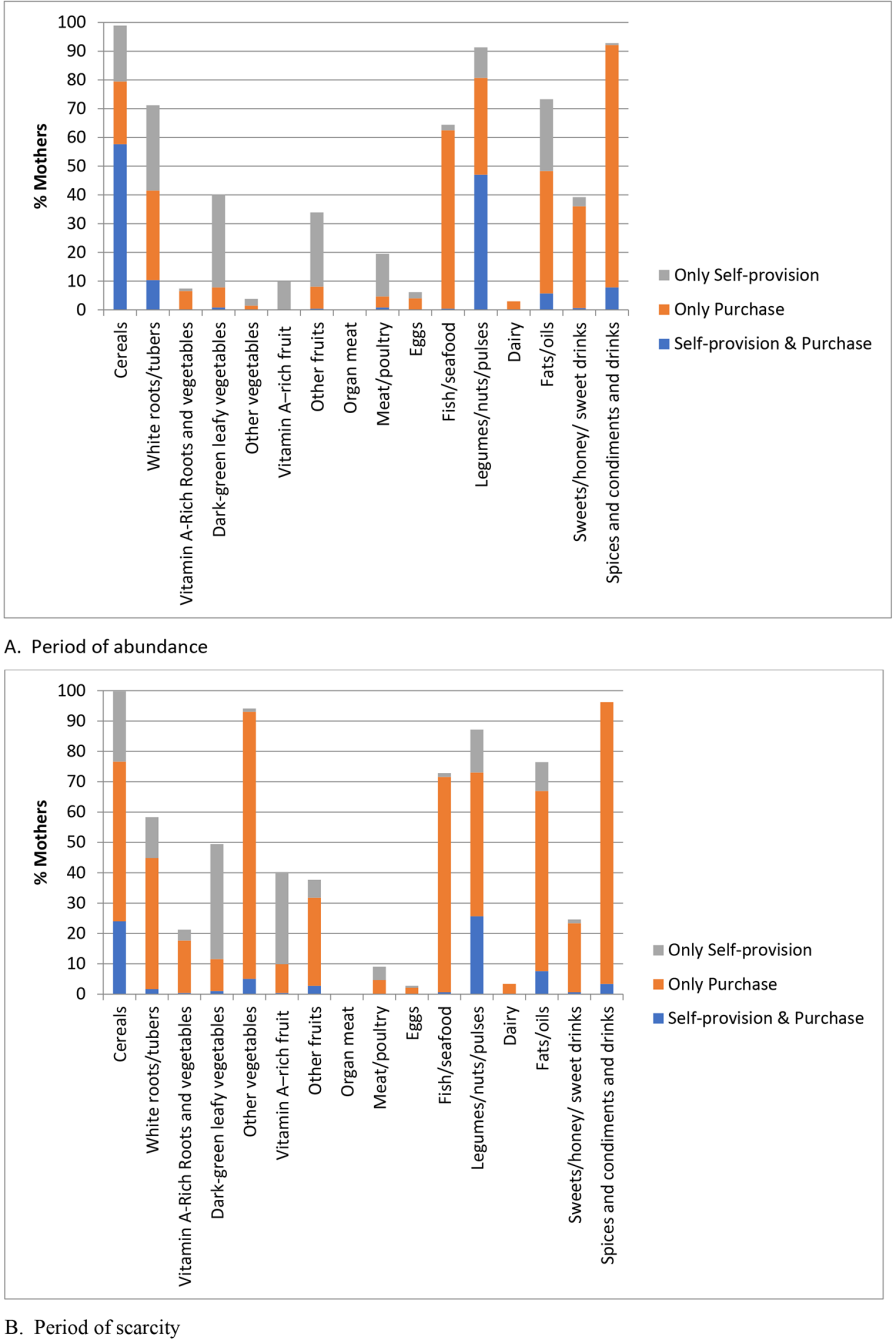
Percentage of mothers that consumed each of the food groups by source, including their combination, in both seasons

An important issue is that mothers obtained foods contributing to the same food group from multiple sources. Figure 1 shows that there is variation in the relative number of mothers that consumed each of the food groups in both periods. For a large percentage of mothers, purchases are a source of foods contributing to most food groups, predominantly during the scarcity period. However, self-provision is a source of foods contributing to certain food groups of high nutritional value during both periods. For two of the most widely consumed food groups, cereals and legumes/nuts/pulses, many mothers sourced them simultaneously through self-provision and purchases in both periods.

### Regression results

Diagnostic tests show the appropriateness of our econometric approach and the soundness of our empirical results (Supplemental Material, Text 1 and Table S4).

Regression results (Table 3) show that mothers in HHs with higher PD consumed a higher level of DD from self-provision, but a lower level of DD from purchases. Mothers from HHs that visited more markets consumed a higher level of DD from purchases.

In terms of the instrumental variables, HHs with larger landholdings maintained higher levels of PD, but this variable had no effect on the number of markets visited. HHs with higher scores in the socioeconomic index visited more markets, but this variable had no effect on PD, as expected.

Among the covariates, travel time to the main market town, a common proxy for access to markets, indicated that HHs located farther away from the main market town have more PD and the respective mothers consumed a higher DD from self-provision but lower DD from purchases. HHs with more sources of income and a higher importance of agricultural income had higher levels of PD, and to a lesser extent visited more markets. However, mothers with a higher importance of agricultural income consumed lower levels of DD from self-provision. Mother’s ethnicity was associated with PD, MP, and DD from purchases, while mother’s education was associated only with the last two variables. Rural HHs located in the urban district had higher PD than those located in the other two districts, while rural HHs located in the semi-urban district visited more markets than those in the other two districts. In terms of seasonality, HHs had lower levels of PD during the abundance period than during the scarcity period. This result simply reflects that a lower number of species are produced during the shorter rainy season compared to those produced during the long rainy season (recall how seasons were defined). The positive association of the abundance period with DD_s_ indicates that while a lower number of species were produced, more species for self-consumption are available after harvest compared to those that still have not yet been harvested (scarcity season), and that there is less need to purchase foods, thus the negative association with DD_p_.

**Table 3:** Regression results

	Production Diversity		Market Participation		Dietary Diversity score from purchases		Dietary Diversity score from self-provision	
	coef.	*p*-value	coef.	*p*-value	coef.	*p*-value	coef.	*p*-value
Production Diversity					**-0.025**	**0.017**	**0.071**	**0.011**
Number of markets					**0.229**	**0.049**	-0.710	0.445
**Instrumental variables**								
Landholdings	**0.136**	**0.002**	-0.001	0.950				
Square of landholdings	**-0.008**	**0.088**	0.001	0.697				
Socioeconomic Index	0.008	0.760	**0.055**	**0.007**				
**Covariates**								
Travel time	**0.014**	**< 0.001**	0.001	0.599	**-0.007**	**0.001**	0.008	0.144
Square of travel time	**0.000**	**< 0.001**	0.000	0.869	**0.000**	**0.019**	0.000	0.464
Mother age	**-0.006**	**0.056**	0.002	0.564	0.000	0.961	0.003	0.539
Mother education	-0.005	0.586	**-0.011**	**0.096**	**0.015**	**0.002**	-0.015	0.222
Mother ethnicity (Aizo)	**0.128**	**0.016**	**0.114**	**0.030**	**-0.087**	**0.014**	0.113	0.240
Family size	**0.028**	**0.001**	-0.001	0.875	0.006	0.271	-0.016	0.219
No. non-agricultural income sources father	**0.070**	**0.006**	**0.051**	**0.029**	0.010	0.663	-0.019	0.774
Agriculture rated very important income source father	**0.416**	**< 0.001**	-0.029	0.478	0.029	0.530	-0.062	0.512
Agriculture rated important income source father	**0.354**	**< 0.001**	0.008	0.863	0.030	0.457	0.037	0.702
No. non-agricultural income source mother	**0.149**	**< 0.001**	**0.043**	**0.080**	0.011	0.567	0.035	0.548
Agriculture rated very important income source mother	**0.280**	**0.000**	0.009	0.829	-0.040	0.376	0.026	0.761
Agriculture rated important income source mother	**0.311**	**0.000**	-0.024	0.551	0.050	0.267	**-0.209**	**0.026**
Urban district	**0.242**	**0.000**	0.025	0.624	0.013	0.720	0.124	0.210
Semi-urban district	0.023	0.646	**0.127**	**0.032**	-0.023	0.595	0.078	0.665
Season (*abundance*)	**-0.436**	**0.000**	-0.021	0.481	**-0.319**	**0.000**	**0.473**	**0.000**
Constant	**0.771**	**< 0.001**	0.060	0.553	**1.750**	**< 0.001**	0.893	0.325

Note: *p*-values are obtained by robust standard-errors and clustered at household level. Numbers in bold indicate statistical significance at least at the 10% level.

## Discussion and policy implications

**Fig. 2:**
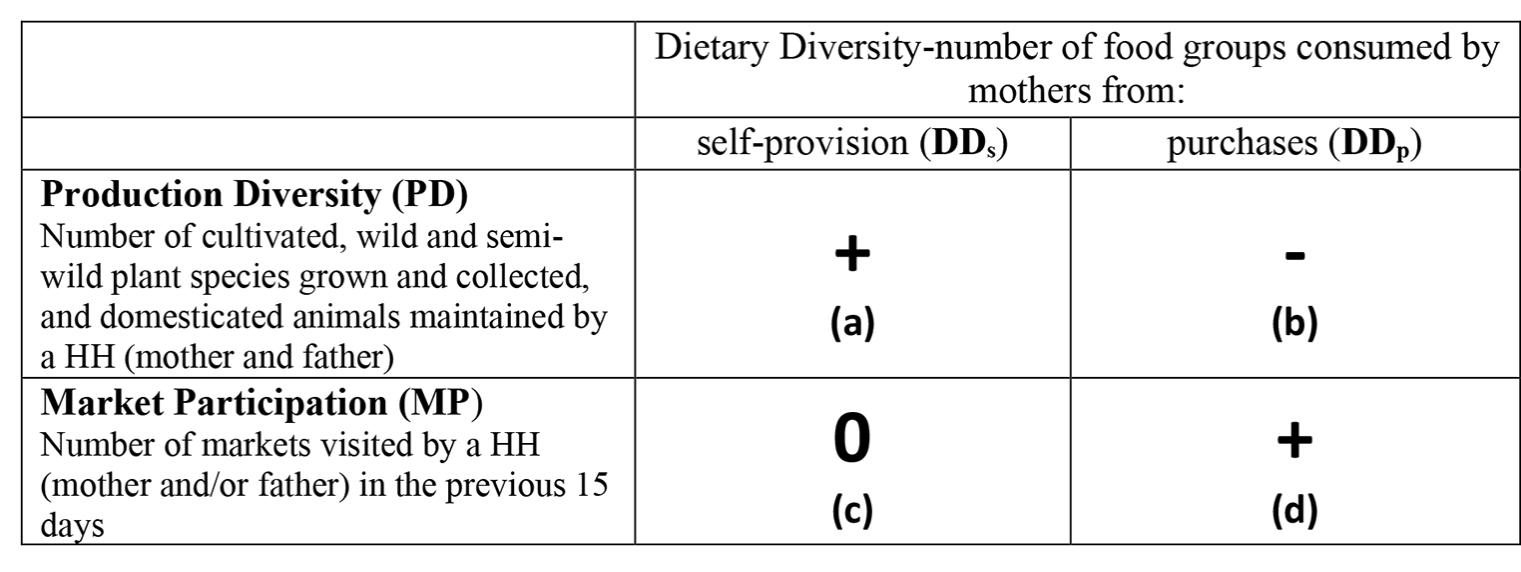
Summary of the direction of the associations found among the variables of interest. (+) indicates a positive and statistically significant association. (-) indicates a negative and statistically significant association. (0) indicates no statistically significant association

The direction of the key associations found among the variables of interest are summarized in Fig. 2. PD is associated positively with DD_S_ as expected (a), but negatively with DD_P_ (b), suggesting that PD lessens the need to purchase diverse foods. MP is positively associated with DD_P_ (d), indicating that visiting more markets contributed to more diversified diets through purchases. In addition, we also showed that visiting more markets is costly in terms of travel time, (i.e., positive correlation between number of markets visited and the log of travel time). These results support the hypothesis that number of markets visited is an indicator of the diversity of foods available in the markets HHs have access to because we have shown that mothers from households who incurred in higher costs to visit more markets consumed higher dietary diversity from purchased foods. Furthermore, this variable is significant after correcting for travel time to the main market in the district, a common variable used to measure market access in many of the studies reviewed in the introduction. The lack of association of visited markets with DD_s_ (c) suggests that the availability of diverse foods in markets has no effect on the diversity of foods sourced from self-provisioning. Therefore, the former does not seem to be a substitute for the latter. Taken together these results indicate that PD is a significant driver of DD, even if it is a more limited source of different foods and food groups than markets. This may be due to the fact that PD may be a cheaper source of diverse foods for HHs and it is under their direct control. As we showed, visiting multiple markets is costly, while PD involves important economies of scope for HHs due to its multiple roles in their livelihoods beyond fulfilling their own food consumption needs. These roles include managing risk (Asfaw et al., 2019; di Falco & Chavas, 2009), optimizing crop production under heterogeneous agro-ecological conditions in rainfed marginal areas (di Falco & Chavas, 2009; Kawa et al., 2015), and providing commercial opportunities in multiple local markets (Devaux et al., 2009; McCord et al., 2015). Therefore, the multiple benefits of PD lessen its cost as a source of foods.

Even if most HHs source many food groups from their own production, with gathering and gifts particularly important for some nutritionally relevant food groups (e.g., dark-green leafy vegetables, vitamin A-rich fruits), most food groups consumed were purchased in markets, as other studies have shown (e.g., Hirvonen & Hoddinott, 2017; Muthini et al., 2020). So, clearly market participation is fundamental for HHs’ access to a variety of food groups that underpin their DD. The finding that market and nonmarket food sources can contribute to the same food groups (e.g., cereals and legumes/nuts/pulses), suggests that their combination may provide some redundancy to HHs’ diets, since redundancy has been defined as “ … the extent to which elements of the systems are replaceable affecting the capacity to absorb the perturbing effect of the disturbance and avoid as much food insecurity as possible” (Tendall et al., 2015, 19). This highlights the important benefits of maintaining multiple food sources.

Our results challenge the studies that argue for policies and interventions that privilege market access and crop specialization over promoting PD for self-consumption as a more effective way to improve HH dietary quality and food security (Dillon et al., 2015; Koppmair et al., 2016; Muthini et al., 2020; Hirvonen & Hoddinott, 2017; Sibhatu et al., 2015; Sibhatu & Qaim, 2018) by showing the complementary roles of market and nonmarket sources of foods resulting from the interactions between PD and market participation. Furthermore, the argument that promoting PD may prevent gains from crop specialization, in turn resulting in income loss and negative nutritional effects (Koppmair et al., 2016; Sibhatu et al., 2015; Sibhatu & Qaim, 2018; Muthini et al., 2020), ignores that crop specialization also may entail costs to rural HHs due to the loss of benefits associated with PD aside from its impacts on DD, such as risk management, adaptation to heterogeneous environments, and more market opportunities (Bellon et al., 2020). In fact, as we have shown in other work under similar conditions, crop diversification actually opens opportunities for generating additional income for rural HHs while providing them with less expensive foods (Bellon et al., 2020). In addition, increased income does not necessarily translate into better nutrition (Herforth & Ahmed, 2015). The relationship between income and nutrition depends on the specific availability, affordability, convenience, and desirability of foods, i.e., the food environment, faced by the HHs (Herforth & Ahmed, 2015). Market and nonmarket sources of foods and the factors that influence them are important components of the food environment of rural HHs and therefore should be considered for policies and interventions to improve nutrition outcomes among rural HHs.

We are not arguing for ignoring interventions promoting market access in favor of strengthening production for self-consumption. On the contrary, our own results show the importance of purchased foods, and thus market participation, as a key source of foods, as well as showing the multiple interactions of HHs with markets. However, we argue for a more holistic approach that recognizes the complementarities between market and nonmarket sources of foods for rural HHs, and thus for interventions and policies that build on them. In particular, we caution against the blanket promotion of interventions that foster crop specialization for income generation in the hope of improving diets and nutrition. Instead, if HHs already maintain PD, a more sensible approach is to build on this diversity, including foods from gathered species. Obviously, this will depend on the context and require targeted interventions. In many cases interventions should focus not on adding additional crops, but on preventing the loss of nutritionally relevant crops already produced, gathered, or shared by rural HHs. For example, the excessive emphasis on promoting a few staple crops, such as maize or rice, has led to a decrease in the production of traditional crops that were important sources of critical micronutrients (Pingali, 2012). Interventions that build on PD also require a better understanding of the structure and function of local markets (Ickowitz et al., 2019). There is a need to go beyond improving the value chains of a few key species and address the diversity of species and foods that are traded in markets. A positive development is the increasing efforts to develop and test methodologies for identifying constraints and bottlenecks in the multiple value chains of nutritious foods to identify and target interventions that link producers and consumers of these foods in specific contexts (Gelli et al., 2020). Given that rural HHs also may sell their production in local markets, and that they may be an important source of highly nutritious but perishable foods, suggests that at the district level, a combination of production diversity with better infrastructure to support market participation is likely to be the best approach for ensuring household access to diverse diets (Ickowitz et al., 2019).

Our study has several limitations. As a case study, its results may only be valid for the specific sample and conditions under which it was implemented. This study is observational and as such causality is not assured. While we identified and addressed endogeneity issues in our model estimation, we cannot completely exclude that the instruments used are correlated with the unobservable determinants of the final outcomes.

## Conclusion

This study has shown that both market and nonmarket sources of foods make important and complementary contributions to the dietary diversity of rural HHs in our study area. Our results suggest that to improve dietary quality and food security among rural HHs under rainfed conditions in similar circumstances to the ones studied here, there is a need for policies and interventions that build on a more holistic approach that recognizes the complementarities between market and nonmarket sources of foods rather than on the blanket promotion of crop specialization for income generation in the hope of improving diets and nutrition. This approach should build on the PD already utilized by rural HHs, and on the ways they interact with markets.

## Electronic supplementary material

Below is the link to the electronic supplementary material.

Supplementary Material 1

Supplementary Material 2
