# Supplementary material for: Combining market and nonmarket food sources provides rural households with more options to achieve better diets in Southern Benin
Source: Food Secur. 2022 Nov 25;15(2):411–22. doi: 10.1007/s12571-022-01320-w (PMC10066082; doi:10.1007/s12571-022-01320-w)
Supplement: Supplementary file 1 — Supplementary Material 1 [file 12571_2022_1320_MOESM1_ESM.docx]

Combining market and nonmarket food sources provides rural households with more options to achieve better diets in Southern Benin

Mauricio R. Bellon^1^, Gervais Ntandou-Bouzitou^2^, Janet E. Lauderdale^3^, Francesco Caracciolo^4^

^1^Swette Center for Sustainable Foods Systems, Arizona State University, Tempe, AZ, United States of America

^2^Food and Agriculture Organization (FAO) of the United Nations, Niamey, Niger

^3^Independent Nutrition Consultant, Independent Nutrition Consultant, Tempe AZ, United States of America

^4^Department of Agricultural Sciences, University of Naples Federico II, Portici (Na), Italy

Address for correspondence: Mauricio R. Bellon, Swette Center for Sustainable Food Systems, Arizona State University, PO Box 878204, Tempe, AZ 85287-8204

**Abstract**

This study examines the relative contributions to dietary diversity of the diversity of plant and domesticated animal species which rural households produce or collect, i.e., nonmarket food sources, versus the diversity of foods purchased in markets. Although opinions differ in the literature as to their relative importance, clarifying how different sources of food contribute to the dietary diversity of rural households in the developing world is important to inform policies and interventions to improve their food security and dietary quality. This case study was carried out among a random sample of 654 rural households in southern Benin during two seasons: when food is plentiful after harvest; and when food is scarcer between harvests. We collected data on crops, wild plants, and domesticated animals utilized by households, the number of markets they visited, and the diet of a mother in the household, with a structured 24-hour food frequency questionnaire. We hypothesize that the number of markets visited is an indicator of the diversity of foods available in the markets they have access to, and thus shows the contribution of markets as food sources. Results support this hypothesis and show that households that produced more plant and domesticated animal species and those that visited more markets had more diversified diets. Obtaining diverse foods from multiple sources provides households with more options to achieve better diets. These results suggest a need for a more holistic approach that recognizes the complementarities between market and nonmarket sources of foods. This approach should build on the diversity of species rural households already utilize, and on the ways they interact with markets.

Keywords: Production diversity, market participation, dietary diversity, Benin, West Africa
