# Supplementary material for: Combining market and nonmarket food sources provides rural households with more options to achieve better diets in Southern Benin
Source: Food Secur. 2022 Nov 25;15(2):411–22. doi: 10.1007/s12571-022-01320-w (PMC10066082; doi:10.1007/s12571-022-01320-w)
Supplement: Supplementary file 2 — Supplementary Material 2 [file 12571_2022_1320_MOESM2_ESM.docx]

Supplemental Material

Supplemental Material, Text 1. Econometric approach

Due to the use of moment conditions, results are asymptotically equivalent to a full information instrumental variables efficient (FIVE) estimator (Bundy and Jorgenson 1971). Results are unbiased from potential sources of endogeneity (i.e., simultaneity, reverse causality) between the outcomes, and specifically PD and MP with DD_S_ and DD_P,_ if valid instrumental variables are used (Heckman and Vytlacil 2005).

Supplemental Material Text 2. Characterization of market types in the study area (based on Honfoga et al. (2018) and direct observation)

(i) Primary markets are local markets that take place once or twice a week. Farmers sell directly to village retailers and small-scale assemblers, mostly in small quantities. Retail consumers, who may also be farmers selling their products, can purchase products from farmers and local traders; the diversity of products offered is limited, mainly to the produce from the villages surrounding the market.

(ii) Rural consumer markets are district-level retail markets that take place every day and have permanent retailers, but also itinerant traders, and on particular days (once or twice a week) farmers may sell wholesale directly there as well. This type is focused on retail consumers; the diversity of products is much larger than in primary markets.

(iii) Rural assembly markets are district-level take place two or three times a week where products from many villages/primary markets are assembled for rural semi-wholesalers, but retail consumers can purchase products as well. They offer more product diversity than primary but less than rural retail markets. This type of market offers more affordable products than rural retails markets.

(iv) Regional markets collect products from assembly markets for urban wholesalers; they take place every day and have permanent traders, as well as itinerant traders, and on particular days of the week (once or twice a week) farmers may sell wholesale directly there as well. They have large product diversity. In some towns rural consumer and regional markets coincide.

Supplemental Material, Text 3. Diagnostic test results

Results from the Durbin–Wu–Hausman test for endogeneity verified presence of endogeneity problems and therefore interdependency in the HHs decision-making process regarding *PD, MP,* and *DD_S_* and *DD_P_*, corroborating the need for using a system of simultaneous equations approach. The empirical soundness of the results is supported by the Hansen-Sargan econometric test of over-identifying restrictions showed the exogeneity of the instruments, while results the test for Weak Instruments (Cameron and Trivedi 2005) show that the selected instruments are relevant (Table S4).


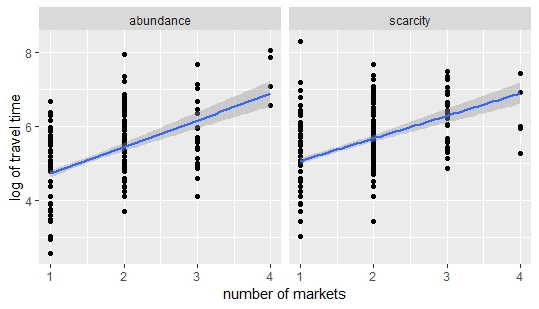


Fig. S1 Relationship between the number of markets visited by HHs and the natural log of the travel time they invested during the abundance period and in the scarcity period (only HHs that visited at least one market).

Table S1. Percentage of HHs attending the different types of markets (number of HHs by district in parenthesis)

|  |  | Abundance | | | | Scarcity | | | | |
| --- | --- | --- | --- | --- | --- | --- | --- | --- | --- | --- |
| Type of market | No. of markets | Urban (115) | Semi-urban (174) | Rural (183) | Total (472) | No. of markets | Urban (115) | Semi-urban (176) | Rural (191) | Total (482) |
| Primary | 13 | 13.9 | 44.3 | 2.7 | 20.8 | 13 | 13.9 | 26.1 | 29.3 | 24.5 |
| Rural assembly | 8 | 5.2 | 33.3 | 10.9 | 17.8 | 9 | 5.2 | 26.7 | 36.6 | 25.5 |
| Rural consumer | 13 | 8.7 | **73.6** | **80.3** | 60.4 | 10 | 8.7 | **32.4** | **100.0** | 61.2 |
| Regional | 8 | **95.7** | 4.0 | 2.7 | 25.8 | 4 | **92.2** | 2.8 | 7.9 | 26.1 |

Table S2. Contribution of different sources of foods consumed by mothers clustered by 16 food groups during period of abundance (number of mothers)

|  | Total (any source) | Self-production | Purchase | Borrow | Gift | Gather |
| --- | --- | --- | --- | --- | --- | --- |
| Cereals | 467 | 360 | 375 | 1 | 15 | 0 |
| White roots/tubers | 336 | 141 | 196 | 1 | 53 | 15 |
| Vitamin A-rich roots and vegetables | 35 | 2 | 31 | 0 | 0 | 2 |
| Dark-green leafy vegetables | 189 | 39 | 37 | 0 | 8 | 109 |
| Other vegetables | 18 | 6 | 7 | 0 | 0 | 5 |
| Vitamin A–rich fruit | 47 | 9 | 0 | 0 | 3 | 37 |
| Other fruits | 160 | 40 | 38 | 1 | 32 | 64 |
| Organ meat | 0 | 0 | 0 | 0 | 0 | 0 |
| Meat/poultry | 92 | 15 | 22 | 0 | 19 | 45 |
| Eggs | 31 | 6 | 19 | 0 | 3 | 1 |
| Fish/seafood | 305 | 3 | 295 | 1 | 1 | 7 |
| Legumes/nuts/pulses | 431 | 199 | 381 | 0 | 46 | 65 |
| Dairy | 14 | 0 | 14 | 0 | 0 | 0 |
| Fats/oils | 346 | 130 | 228 | 0 | 14 | 1 |
| Sweets/honey/ sweet drinks | 185 | 2 | 170 | 1 | 9 | 7 |
| Spices and condiments and drinks | 438 | 14 | 435 | 0 | 27 | 0 |
| Mothers who consumed food groups from | 472 | 423 | 470 | 5 | 170 | 240 |
| Average number of food groups per mother groups consumed from | 6.56 | 2.05 | 4.76 | 0.01 | 0.49 | 0.76 |
|  | (1.75) | (1.32) | (1.69) | (0.10) | (0.77) | (0.92) |

Table S3. Contribution of different sources of foods consumed by mothers clustered by 16 food groups period of scarcity (number of mothers)

|  | Total (any source) | Self-production | Purchase | Borrow | Gift | Gather |
| --- | --- | --- | --- | --- | --- | --- |
| Cereals | 474 | 217 | 364 | 0 | 12 | 0 |
| White roots/tubers | 277 | 66 | 213 | 0 | 6 | 0 |
| Vitamin A-rich roots and vegetables | 101 | 10 | 84 | 0 | 3 | 6 |
| Dark-green leafy vegetables | 235 | 79 | 55 | 0 | 12 | 96 |
| Other vegetables | 447 | 20 | 442 | 0 | 6 | 3 |
| Vitamin A–rich fruit | 191 | 59 | 47 | 0 | 6 | 81 |
| Other fruits | 179 | 16 | 151 | 0 | 13 | 14 |
| Organ meat | 0 | 0 | 0 | 0 | 0 | 0 |
| Meat/poultry | 43 | 7 | 22 | 0 | 9 | 5 |
| Eggs | 13 | 3 | 10 | 0 | 0 | 0 |
| Fish/seafood | 346 | 1 | 340 | 0 | 6 | 2 |
| Legumes/nuts/pulses | 414 | 155 | 347 | 0 | 27 | 11 |
| Dairy | 16 | 0 | 16 | 0 | 0 | 0 |
| Fats/oils | 363 | 75 | 318 | 0 | 0 | 0 |
| Sweets/honey/ sweet drinks | 117 | 0 | 111 | 0 | 9 | 0 |
| Spices and condiments and drinks | 457 | 2 | 457 | 1 | 14 | 0 |
| Mothers who consumed food groups from | 475 | 329 | 475 | 1 | 91 | 165 |
| Average number of food groups per mother groups consumed from | 7.73 | 1.49 | 6.27 | 1.00 | 0.27 | 0.46 |
|  | (1.71) | (1.47) | (1.86) | na | (0.70) | (0.71) |

Table S4. Diagnostic tests for endogeneity, instrument validity and relevance

| Durbin–Wu–Hausman test for endogeneity^a^ |  |
| --- | --- |
| *PD* | F(1,865): 2.82 (p = 0.09) DDS; F(1,865): 2.42 (p = 0.12) DD_P_ |
| *MP* | F(1,865): 0.60 (p = 0.44) DDS; F(1,865): 0.94 (p = 0.33) DD_P_ |
| Hansen-Sargan overidentification statistic^b^: |  |
|  | Hansen's J chi^2^(2): 2.763 (p = 0.251) |
| Test for weak instrument^c^ |  |
| *PD* | F(3,864): 23.05 (p<0.001) |
| *MP* | F(3,864): 2.61 (p = 0.050) |

^a^ Small p-values indicate inconsistency of OLS, H0: E(u|x) = 0.

^b^ Small p-values indicate instrument inconsistency, H0: E(z|u) = 0.

^c^ Small p-values indicate instruments relevance, H0: E(z|x) = 0

**References**

Cameron, A.C., &Trivedi, P.K., (2005). *Microeconometrics: methods and applications*. New York; Cambridge University Press.

Heckman, J.J., & Vytlacil, E.J. (2005). Structural equations, treatment effects, and econometric policy evaluation*. Econometrica*, 73, 669-738.

Honfoga, B. G., Ntandou-Bouzitou, G., Vodouhe R. S., Bellon, M. R., and Hounhouigan, J. D. (2018). Assessing the role of market integration in the consumption of traditional food in Benin: a joint price instability coefficient and diet composition approach. *Agricultural and Food Economics*, 6, 2(2018) <https://doi.org/10.1186/s40100-018-0097-1>
